# Genetic diversity, phylogeography and molecular clock of the *Lutzomyia longipalpis* complex (Diptera: Psychodidae)

**DOI:** 10.1371/journal.pntd.0006614

**Published:** 2018-07-05

**Authors:** Angélica Pech-May, Janine M. Ramsey, Raúl E. González Ittig, Magali Giuliani, Pablo Berrozpe, María G. Quintana, Oscar D. Salomón

**Affiliations:** 1 Instituto Nacional de Medicina Tropical, Ministerio de Salud de la Nación, CONICET, Puerto Iguazú, Misiones, Argentina; 2 Instituto Nacional de Salud Pública / Centro Regional de Investigación en Salud Pública, Tapachula, Chiapas, México; 3 Instituto de Diversidad y Ecología Animal (IDEA), CONICET-Facultad de Ciencias Exactas, Físicas y Naturales, Universidad Nacional de Córdoba, Córdoba, Argentina; 4 Universidad Nacional de Tucumán- CONICET, Instituto Superior de Entomología, FCNeIML, San Miguel de Tucumán, Argentina; Saudi Ministry of Health, SAUDI ARABIA

## Abstract

**Background:**

The *Lutzomyia longipalpis* complex has a wide but discontinuous distribution in Latin America, extending throughout the Neotropical realm between Mexico and northern Argentina and Uruguay. In the Americas, this sandfly is the main vector of *Leishmania infantum*, the parasite responsible for Visceral Leishmaniasis (VL). The *Lu*. *longipalpis* complex consists of at least four sibling species, however, there is no current consensus on the number of haplogroups, or on their divergence. Particularly in Argentina, there have been few genetic analyses of *Lu*. *longipalpis*, despite its southern expansion and recent colonization of urban environments. The aim of this study was to analyze the genetic diversity and structure of *Lu*. *longipalpis* from Argentina, and to integrate these data to re-evaluate the phylogeography of the *Lu*. *longipalpis* complex using mitochondrial markers at a Latin American scale.

**Methodology/Principal findings:**

Genetic diversity was estimated from six sites in Argentina, using a fragment of the ND4 and the 3´ extreme of the cyt *b* genes. Greatest genetic diversity was found in Tartagal, Santo Tomé and San Ignacio. There was high genetic differentiation of *Lu*. *longipalpis* in Argentina using both markers: ND4 (F_ST_ = 0.452, *p* < 0.0001), cyt *b* (F_ST_ = 0.201, *p* < 0.0001). Genetic and spatial Geneland analyses reveal the existence of two primary genetic clusters in Argentina, cluster 1: Tartagal, Santo Tomé, and San Ignacio; cluster 2: Puerto Iguazú, Clorinda, and Corrientes city. Phylogeographic analyses using ND4 and cyt *b* gene sequences available in GenBank from diverse geographic sites suggest greater divergence than previously reported. At least eight haplogroups (three of these identified in Argentina), each separated by multiple mutational steps using the ND4, are differentiated across the Neotropical realm. The divergence of the *Lu*. *longipalpis* complex from its most recent common ancestor (MRCA) was estimated to have occurred 0.70 MYA (95% HPD interval = 0.48–0.99 MYA).

**Conclusions/Significance:**

This study provides new evidence supporting two *Lu*. *longipalpis* genetic clusters and three of the total eight haplogroups circulating in Argentina. There was a high level of phylogeographic divergence among the eight haplogroups of the *Lu*. *longipalpis* complex across the Neotropical realm. These findings suggest the need to analyze vector competence, among other parameters intrinsic to a zoonosis, according to vector haplogroup, and to consider these in the design and surveillance of vector and transmission control strategies.

## Author summary

The *Lutzomyia longipalpis* complex has a wide but discontinuous distribution in Latin America, extending throughout the Neotropical realm between Mexico and northern Argentina and Uruguay. In the Americas, this sandfly is the main vector of *Leishmania infantum*, the parasite responsible for Visceral Leishmaniasis (VL). The *Lu*. *longipalpis* complex is composed of at least four sibling species, although there is no current consensus on the number of haplogroups, or their divergence. In Argentina, little is known about the complex population structure. Therefore, the aim of this study was to analyze the diversity and genetic structure of *Lu*. *longipalpis* from Argentina and subsequently to analyze the complex phylogeography at a Latin American scale, using two mitochondrial markers. Greatest genetic diversity was found in Tartagal, Santo Tomé and San Ignacio. Two genetic clusters and three *Lu*. *longipalpis* haplogroups were identified from the six sites in Argentina. Phylogeographic analyzes using ND4 and cyt *b* gene sequences and those from across the Neotropical realm registered in GenBank, suggest greater divergence than previously reported. At least eight haplogroups are differentiated using the ND4, each separated by multiple mutational steps. *Lu*. *longipalpis* complex divergence from its most recent common ancestor (MRCA) was estimated at mid Pleistocene, 0.70 MYA (95% HPD interval = 0.48–0.99 MYA).

## Introduction

Visceral leishmaniasis (VL) is a parasitic disease caused in the American continent, by *Leishmania infantum* (syn. *Le*. *chagasi*). VL has an estimated global incidence of 500,000 cases and 59,000 deaths per year [1]. Human cases of VL in Latin America are reported in 12 countries, while Argentina reports an expanding epidemiological scenario [2]. The first autochthonous VL cases in both humans and canines in Argentina were reported in 2006, in the city of Posadas, Misiones [3]. Currently, the country has reported 106 human cases of VL, with 7.7% mortality, between 2006 and 2016 [4]. Misiones is the province with most human VL cases; the age group with highest incidence is children 0–15 yrs [5]. In Argentina, the main vector of *Le*. *infantum* is *Lutzomyia longipalpis* (Lutz & Neiva) [3]. This phlebotomine was first reported in 1951 in Candelaria, and later in 2000, in Corpus, both in Misiones [6]; there were no VL cases reported at either time. The first autochthonous VL human cases with parasite presence confirmed were reported from Posadas, in 2006 [3]. At present, the vector has dispersed to southern Argentina while also expanding in the north. Currently, *Lu*. *longipalpis* is also reported in Corrientes, Entre Ríos, Chaco, Formosa, and Salta provinces [5, 7–10].

*Lutzyomia longipalpis* is currently considered a species complex, despite a broad yet discontinuous distribution within the Neotropical realm, from Mexico to the north of Argentina and Uruguay [9, 11]. Recent studies using ecological niche models report that anticipated distributional shifts of *Lu*. *longipalpis* vary by region, although greater projected landscape fragmentation and anthropic modifications will not significantly affect model projections [12, 13]. This sandfly is adapted to a variety of habitats in tropical regions, from rocky, arid, semi-arid, to very humid, and forested [11, 14]. Throughout the species´ distribution, different patterns of genetic divergence and evidence for cryptic species have been reported [15–20]. Mangabeira [21] was the first to evidence ecological and morphological differences between populations of *Lu*. *longipalpis* collected in Pará and Ceará states, Brazil. These males had one pair of pale tergal spots on abdominal tergite IV (phenotype 1S), and another additional pair on tergite III (phenotype 2S). Other studies in *Lu*. *longipalpis* have reported sympatry of the differentiated phenotypes [22, 23], isoenzyme variability [19, 23–26], differences in sex pheromones [27–29], variation in the salivary peptide, maxadilan [30, 31], differences in male copulation songs [29], wing morphometric variation [24], and genetic variability and divergence using multiple genetic markers [16, 18, 19, 28, 29, 32–34]. Mitochondrial genes are considered good tools for population genetics and phylogeography due to their abundance, little or no recombination and a haploid mode of inheritance [35]. Fragments of the nicotinamide dinucleotide dehydrogenase subunit 4 (ND4) and the 3' region of cytochrome *b* (cyt *b*) are highly variable at the inter and intra-specific level in phlebotomine sandflies [16, 36–39]. They have been successfully used to analyze genetic diversity, population genetics, and phylogeography of *Lu*. *longipalpis* [16, 20, 37, 40–42], as well as in other New World sandfly species e.g. *Lu*. *cruciata* (Coquillett) [43], *Lu*. *anduzei* (Rozeboom) [44], *Lu*. *olmeca olmeca* (Vargas & Díaz-Nájera) [45] and old world sandfly species e.g. *Phlebotomus papatasi* (Scopoli) [38, 39], *Phlebotomus ariasi* (Tonnoir) [46] and *Sergentomyia* (*Sintonius*) *clydei* (Sinton) [47]. Genetic diversity, genetic differentiation, and sandfly speciation have been associated with multiple factors, such as latitude or altitude, distance between populations, habitat modifications, anthropogenic landscape fragmentation, vegetation type, geographic barriers (rivers, mountains), host communities, and host species turnover. These factors reduce sandfly dispersal capacity thereby giving rise to isolated populations, loss of genetic diversity, and increasing differentiation among populations [20, 39–41, 43, 44, 46, 48–51].

There are few studies on the genetic status of *Lu*. *longipalpis* in Argentina, although it is clear that the species is expanding southward and is colonizing urban environments [52]. Salomón *et al*. [33] using the “per” gene, found high genetic differentiation between *Lu*. *longipalpis* from Posadas (Misiones province, Argentina) and the north and southeast regions of Brazil, suggesting they might represent another sibling species. In contrast, males from Argentina secrete the same male pheromone as those from Paraguay [53] and other populations from Brazil [27, 29, 54]. It is therefore important to be able to identify and differentiate haplogroups, since they may differ in importance as diseases vectors. Haplogroups may differ in terms of vector competence, vector capacity, or other epidemiological aspects [55], or the clinical expression of disease, based on maxadilan salivary content [31]. Although at least four sibling species have been proposed for the *Lu*. *longipalpis* complex [16, 18, 19, 56, 57], there is insufficient evidence currently for a general consensus on the exact number of haplogroups, or their divergence. The aim of this study was to analyze the genetic diversity and structure of *Lu*. *longipalpis* from Argentina, and to integrate these data to re-evaluate the phylogeography of the *Lu*. *longipalpis* complex using mitochondrial markers at a Latin America scale.

## Methods

### Study area and taxonomic identification

**Fig 1:**
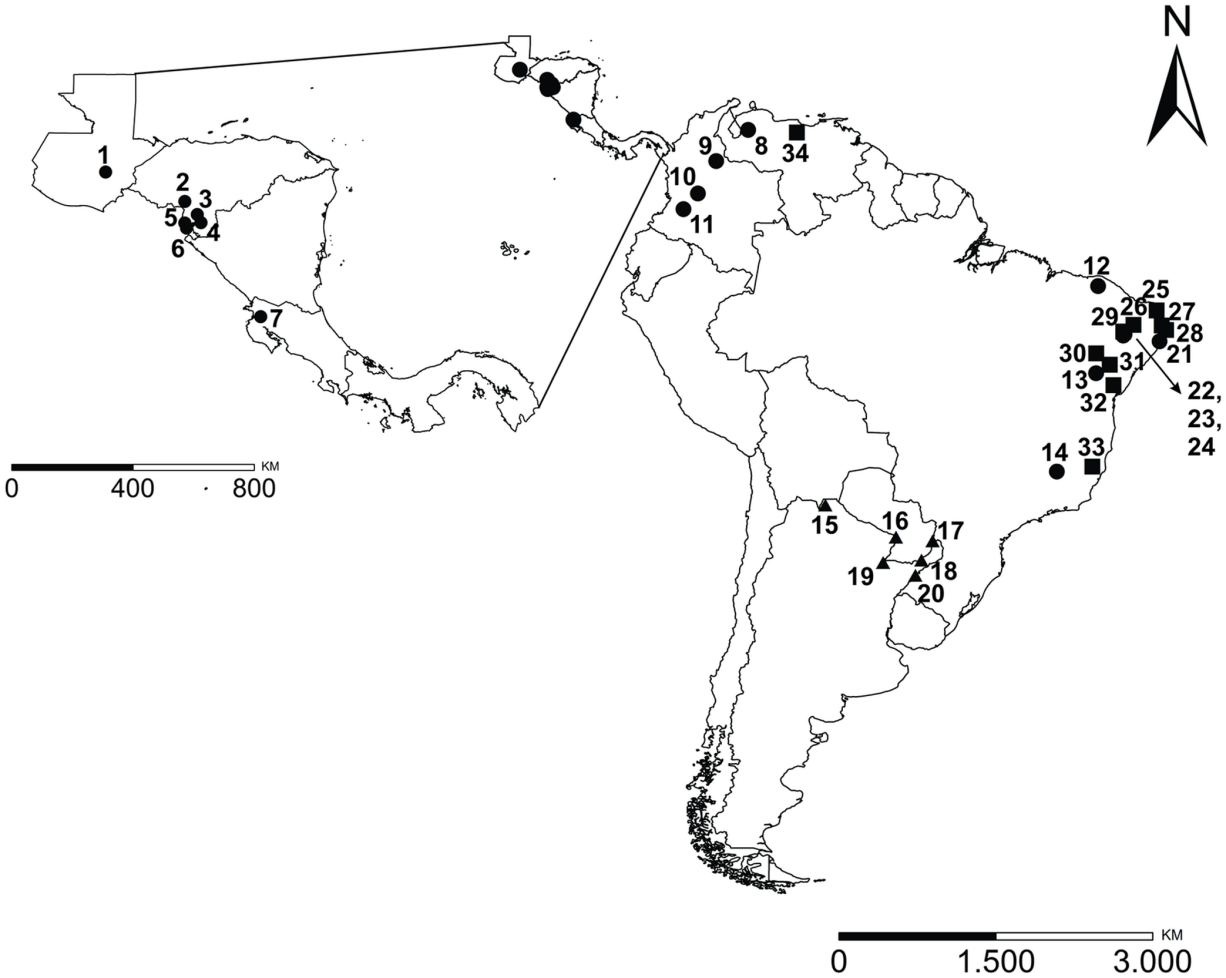
Geographic distribution of *Lutzomyia longipalpis* complex specimens analyzed. Sampling sites for Argentinian specimens for which there are sequences for both gene fragments (triangles), for those sequences analyzed from GenBank for ND4 [16, 42] (circles), and those from GenBank for cyt *b* sequences [20, 37, 40, 41] (square). This map was generated using QGIS v.2.6.1 [61].

*Lutzomyia longipalpis* was collected in six sites in Argentina by the Leishmaniasis Research Network (REDILA). Four of these localities have reported human VL cases (Puerto Iguazú, San Ignacio, Santo Tomé, and Corrientes city), one has reported only canine cases (Clorinda), and one has not reported any VL cases to date (Tartagal) (Fig 1, S1 Table). Clorinda and Corrientes city are located in the humid Chaco ecoregion, which has a warm subtropical climate. Puerto Iguazú and San Ignacio are located in the Paranense forest ecoregion with a humid subtropical climate, and Santo Tomé is found in grassland and forest ecoregion also in a humid subtropical climate. Tartagal is located in the Yunga forest ecoregion with a subhumid warm climate [58]. Sandfly collections were carried out using REDILA-BL light traps [59], and specimens were preserved in 90% ethanol and transported to the laboratory. The last three segments of males were dissected for taxonomic identification, while the rest of the insects were used for molecular analyses. The dissected segments were clarified with lacto-phenol and mounted on slides, and identified using optical microscopy using the Galati taxonomic key [60].

### DNA extraction, amplification and sequencing

Genomic DNA was extracted from individual sandflies using DNA Puriprep-S kit Highway (Inbio, Argentina), according to manufacturer’s instructions. The ND4 fragment was amplified using primers reported by Soto *et al*. [16]. PCR reactions were carried out in 50 μl volumes, containing 25 μl of Gotaq green Master Mix (Promega, USA), 50 pmol/μl of each primer, 2.5 mM of MgCl_2_ and 50 ng of template DNA. Amplification conditions were: 95°C for 5 min, followed by 35 cycles of 95°C (30 sec), 48°C (45 sec), 72°C (1 min) and a final elongation step at 72°C for 10 min. The 3´end of the cyt *b* fragment was amplified using primers and amplification conditions reported by Hodgkinson *et al*. [40, 41] and the PCR was performed according to Pech May *et al*. [43]. PCR products were separated using 1.5% agarose gel electrophoresis, stained with 0.5 μg/ml Sybr Green (Invitrogen, USA), and visualized under UV light. PCR products were purified by HW DNA Puriprep-GP kit (Inbio, Argentina), and sequenced using both forward and reverse primers in a CEQ 2000XL automated sequencer (Beckman Coulter, USA).

### Genetic analyses of Argentinian populations

Forward and reverse sequences from all samples were used to generate consensus sequences and were manually aligned and edited using MEGA v.7 [62]. All haplotypes were deposited in GenBank (accession numbers for the ND4 fragment: MH166358- MH166392; accession numbers for the cyt *b* fragment: MH166339- MH166356). Intra-population and global genetic diversity was analyzed using the number of mutations (*η*), the number of segregating sites (*S*), the number of unique sites (*Su*), the mean number of pairwise differences (*k*), haplotype number (*Nh*), haplotype diversity (*Hd*), nucleotide diversity (*π*), and the nucleotide polymorphism index (*θ*), using DnaSP v.5.10 [63]. Neutrality tests based on Tajima’s D and Fu’s Fs [64, 65] were calculated based on segregating sites, using the same software. In addition, the mismatch distribution test was also analyzed using ARLEQUIN v.3.5 [66]. The goodness-of-fit of the observed data to a simulated expansion model was tested using both Harpending’s raggedness index (*r*) [67] and the sum of squares deviations (*SSD*), using 10,000 replicates. Molecular variance (AMOVA) was used to evaluate population genetic differentiation, using 10,000 random permutations in ARLEQUIN v.3.5. The *p* values of pairwise F_ST_ were adjusted using the Holm-Bonferroni sequential correction “*p*´” [68, 69]. Association between genetic and geographic distance was analyzed using a Mantel test [70], implemented in a trial version of XLTAT (https://www.xlstat.com). Linear geographic distances between sites were estimated using QGIS v.2.6.1 [61].

ND4 and cyt *b* fragments were concatenated and analyzed using GENELAND package in R to infer the number of *Lu*. *longipalpis* genetic clusters among Argentinian sites [71–74]. This program uses a spatial statistical model and Markov chain Monte Carlo sampling with GPS coordinates to estimate the number of populations or genetic clusters (*K*). Preliminarily, we estimated the number of *K* from 1 to 10, using 10 million MCMC iterations and 1,000 thinnings. Five independent runs with fixed “*K”* were run (to avoid ghost populations), assuming an uncorrelated allelic frequency and spatial model. For each run, the posterior probability (PP) of subpopulation membership was computed for each pixel of the spatial domain (100 x 100 pixels), using a burn-in of 1,000 iterations. Variation among and within populations of genetic clusters was analyzed using 10,000 random permutations for the AMOVA in ARLEQUIN v.3.5.

### Phylogeographic analyses: Network haplotypes, phylogenetic inferences, and divergence times

Unique *Lu*. *longipalpis* haplotypes generated herein from Argentina, ND4 gene sequences from GenBank (AF293027—AF293054; AY870836—AY870863 [16, 42]), as well as cyt *b* gene sequences from GenBank (AF448542; AF468979—AF468999; AF480170—AF480181; EF107662—EF107666; HM030727 [20, 37, 40, 41]) were used in analyses (Fig 1, S1 Table). The relationship among haplotypes using ND4 and cyt *b* sequences was evaluated by constructing independent median-joining haplotype networks implemented in Network v.4.6 [75]. The jModeltest v.2 [76] was used to select the best-fitting model of evolution using the Bayesian Information Criterion (BIC). The Bayesian inference (BI) was generated using Mr. Bayes v.3.2 [77]. Posterior probabilities of phylogenetic trees were estimated using 10 million generations (sampled every 1,000 generations) and four Metropolis-coupled Markov chain Monte Carlo (MCMC) to allow adequate time and mixing for convergence. The first 25% of sampled trees were considered as burn in. The consensus tree was visualized using FigTree v.1.4. Sequences of *Migonemyia migonei* (access numbers, ND4: MH166393, cyt *b*: MH166357) and *Phlebotomus papatasi* (access number, ND4 and cyt *b*: KR349298) were used as outgroups.

Divergence times among haplogroups were estimated using BEAST v.1.8.4 [78]. We calibrated using the divergence rate reported by Esseghir *et al*. [36], and corrected as suggested by Ho *et al*. [79], assuming 0.105 substitution/site/million years. An uncorrelated lognormal relaxed-clock model was used to allow rate variation among branches and the coalescent exponential growth option. Convergence of the MCMC chains was checked using TRACER v.1.6, with 200 as the minimum effective sample size. The length of the runs were: a) 100 million generations for the ND4 fragment, and b) 500 million generations for the cyt *b* fragment; sampling was every 10,000 and 50,000 generations, respectively. The first 10% of the samples were discarded as burn-in and a maximum clade credibility tree, reflecting divergence times and their 95% highest posterior densities, was estimated using TreeAnotator of the BEAST package and visualized using FigTree v.1.4.

Nucleotide divergence (*Da*) for the haplogroups was estimated based on the number of net nucleotide substitutions, using the Jukes and Cantor (JC) correction [80, 81] with DnaSP v.5.1.0. The pairwise F_ST_ comparison between haplogroups was performed using 10,000 random permutations in ARLEQUIN v.3.5. The *p* values were adjusted using a Holm-Bonferroni sequential correction “*p*´” [68, 69]. The single sequence from Venezuela was not included in these latter analyses.

## Results

### *Lu*. *longipalpis* genetic diversity in Argentina using the ND4 fragment

Seventy-three specimens of *Lu*. *longipalpis* were sequenced from six Argentinian sites using a 618 bp fragment of the ND4 gene (Table 1A). This fragment had 68 polymorphic nucleotides (11%) and the A-T composition was 73.3%. A total of 35 haplotypes were identified, with a range of 4–14 haplotypes per site. Globally, haplotype diversity was high (*Hd* ± SD = 0.858 ± 0.039), while nucleotide diversity and polymorphism indices were low (*π* ± SD = 0.014 ± 0.001 and *θ* ± SD = 0.022 ± 0.002, respectively). Populations from San Ignacio, Santo Tomé, and Tartagal had highest genetic diversity (Table 1A). The global neutrality tests were, in general, not significant, except for Corrientes city, which was negative and significant for both tests (Tajima *D* = -1.897; *Fs* = -3.724), consistent with population expansion. This result agrees with the mismatch distributions which are unimodal (*SSD* = 0.0003, *p* = 0.979; *r* = 0.019, *p* = 0.992). Population expansion based on the mismatch distribution was also found in Puerto Iguazú (*SSD* = 0.02, *p* = 0.43; *r* = 0.089, *p* = 0.705) and San Ignacio (*SSD* = 0.098, *p* = 0.28; *r* = 0.217, *p* = 0.273). In the latter site, the distribution was multimodal, and hence few individuals shared each haplotype. In contrast, in Tartagal with no indication of population expansion (*SSD* = 0.038, *p* = 0.007; *r* = 0.062, *p* = 0.04), there was a multimodal distribution indicating demographic equilibrium. Analyses for Santo Tomé and Clorinda were inconclusive (*SSD* = 0.966, *p* = 0; *r* = 0.051, *p* = 1; *SSD* = 0.297, *p* = 0.049; *r* = 1.11, *p* = 0.057, respectively) (S1 Fig).

**Table 1:** Genetic diversity indices for *Lu*. *longipalpis* populations from Argentina with the fragments ND4 and 3´cyt *b*.

Molecular marker	Indices	Clorinda	Corrientes	Puerto Iguazú	San Ignacio	Santo Tomé	Tartagal	Global
**A) ND4 618 bp**	N	4	27	10	6	10	16	73
	*h*	5	14	4	22	27	44	69
	*S*	5	13	4	22	27	44	68
	*SU*	613	605	614	595	591	574	550
	*K*	2.66	1.47	1.33	8.13	10.71	12.7	8.65
	*Nh*	4	9	4	5	8	14	35
	*Hd* ± SD	1 ± 0.177	0.68 ± 0.09	0.71 ± 0.11	0.93 ± 0.12	0.93 ± 0.07	0.98 ± 0.02	0.85 ± 0.03
	*π* ± SD	0.004 ± 0.001	0.002 ± 0.0005	0.002 ± 0.0004	0.01 ± 0.004	0.01 ± 0.003	0.02 ± 0.001	0.01 ± 0.001
	*θ* ± SD	0.004 ± 0.001	0.005 ± 0.001	0.002 ± 0.001	0.01 ± 0.003	0.01 ± 0.002	0.021 ± 0.003	0.02 ± 0.002
	Fu´s *Fs* test	-1.41	-3.72***	-0.27	0.47	-0.15	-3.002	-1.35
	Tajima´s Test *D*	-0.21	-1.89*	-0.21	-0.97	0.58	-0.17	-1.27
**B) 3´ Cyt *b* 261 bp**	N	4	26	12	6	12	16	76
	*h*	1	2	2	6	11	16	20
	*S*	1	2	2	6	11	16	20
	*SU*	260	259	259	255	250	245	241
	*K*	0.5	1.03	1.06	2	2.78	3.8	2.34
	*Nh*	2	2	2	3	7	12	18
	*Hd* ± SD	0.500 ± 0.26	0.51 ± 0.03	0.53 ± 0.07	0.60 ± 0.21	0.83 ± 0.10	0.96 ± 0.03	0.77 ± 0.03
	*π* ± SD	0.002 ± 0.001	0.003 ± 0.0002	0.004 ± 0.0005	0.007 ± 0.004	0.01 ± 0.002	0.01 ± 0.001	0.008 ± 0.0009
	*θ* ± SD	0.002 ± 0.002	0.002 ± 0.001	0.002 ± 0.001	0.01 ± 0.004	0.01 ± 0.004	0.01 ± 0.004	0.01 ± 0.003
	Fu´s *Fs* test	0.17	3.31	2.53	1.01	-1.56	-5.59**	-0.01
	Tajima´s Test *D*	-0.61	2.08*	1.75	-1.36	-0.97	-1.27	-1.27

***N*: number of sequences; *h* number of mutations; *S*: number of segregating sites; *Su*: number of unique sites; *k*: mean number of pairwise differences; *Nh*: number of haplotypes; *Hd*: haplotype diversity; *π*: nucleotide diversity; *θ*: nucleotide polymorphism index. SD: standard deviation. Neutrality tests: Fu´s *Fs* and Tajima´s *D*. Statistical significance:** ***p < 0.0001 **p < 0.02; * *p* < 0.05

The global genetic differentiation of *Lu*. *longipalpis* from Argentina using the ND4 fragment is high (F_ST_ = 0.452, *p* < 0.0001), although most of the genetic differentiation was within populations (54.8%). Greatest genetic difference was between San Ignacio and Corrientes city, with an F_ST_ of 0.86 (*p* < 0.0001) (Table 2). There was no evidence for genetic isolation associated with geographic distance (*r* = 0.24, *p* = 0.37).

**Table 2:** Pairwise F_ST_ genetic comparisons between *Lu*. *longipalpis* populations from Argentina. Below diagonal from ND4 fragment, above diagonal from 3´ cyt *b* fragment.

	**Clorinda**	**Corrientes**	**Puerto Iguazú**	**San Ignacio**	**Santo Tomé**	**Tartagal**
**Clorinda**		0.188	0.04	0.336*	-0.23	-0.199
**Corrientes**	0.07		-0.033	*0*.605***	0.242**	*0*.285***
**Puerto Iguazú**	0.038	0.076*		*0*.51**	0.08	0.125*
**San Ignacio**	0.703**	*0*.86***	*0*.791***		0.335**	0.308**
**Santo Tomé**	0.138	*0*.381**	0.207	0.357**		-0.017
**Tartagal**	*0*.368**	*0*.568***	*0*.429***	*0*.3***	0.067	

**Significant differentiation** ***p < 0.0001, **p < 0.02, * *p* < 0.05. **Bold italics indicate *p*´ significant values using a Holm-Bonferroni sequential correction: ND4,** *p*´ < 0.0151 (equal *p* < 0.00168)**; 3´cyt b,** *p*´ < 0.0399 (equal *p* < 0.00307)**.**

### *Lu*. *longipalpis* genetic diversity in Argentina using the cyt *b* fragment

Seventy-six specimens of *Lu*. *longipalpis* were sequenced from all six sites in Argentina, using the 261 bp fragment of the cyt *b* gene (Table 1B). This fragment had 20 polymorphic nucleotides (7.66%), and the A-T composition was 76.3%. The cyt *b* fragment identified 18 haplotypes, ranging from 2 to 12 per site. Globally, haplotype diversity was high (*Hd* ± SD = 0.772 ± 0.036), similar to that for the ND4 fragment, as were low nucleotide diversity and nucleotide polymorphism index (*π* ± SD = 0.008 ± 0.0009 and *θ* ± SD = 0.015 ± 0.003, respectively). San Ignacio, Santo Tomé and Tartagal had highest cyt *b* diversity (Table 1B), although the global neutrality test was not significant. The Tajima test was significant for Corrientes city, but with a positive value (*D* = 2.083), probably due to the low number of haplotypes, suggesting bottleneck events. The mismatch analysis for this site was inconclusive (*SSD* = 0.196, *p* = 0.145, *r* = 0.767, *p* = 0.016). In contrast, in Tartagal, Fu’s *Fs* was negative and significant (*Fs* = -5.598; *p* < 0.0001), consistent with population expansion or selective sweep/hitch-hiking. This latter also agrees with the mismatch distribution analysis (*SSD* = 0.012, *p* = 0.481; *r* = 0.033, *p* = 0.761). Population expansions based on mismatch distributions were also present in Clorinda (*SSD* = 0.021, *p* = 0.655; *r* = 0.25, *p* = 0.93), San Ignacio (*SSD* = 0.089, *p* = 0.405; *r* = 0.204, *p* = 0.74), and Santo Tomé (*SSD* = 0.037, *p* = 0.302; *r* = 0.135, *p* = 0.228). Despite the previous for San Ignacio and Santo Tomé, they had multimodal distributions with pronounced peaks, suggesting that few individuals share each haplotype. There were inconclusive results from Puerto Iguazú (*SSD* = 0.202, *p* = 0.14; *r* = 0.783, *p* = 0.034) (S2 Fig). There was high genetic differentiation (F_ST_ = 0.201, *p* < 0.0001), principally within populations (79.84%). Greatest genetic difference was between San Ignacio and Corrientes city (F_ST_ = 0.605, *p* < 0.0001) (Table 2). Again, there was no evidence for genetic isolation associated with geographic distance (*r* = 0.49, *p* = 0.06).

### Population clusters in Argentina

**Fig 2:**
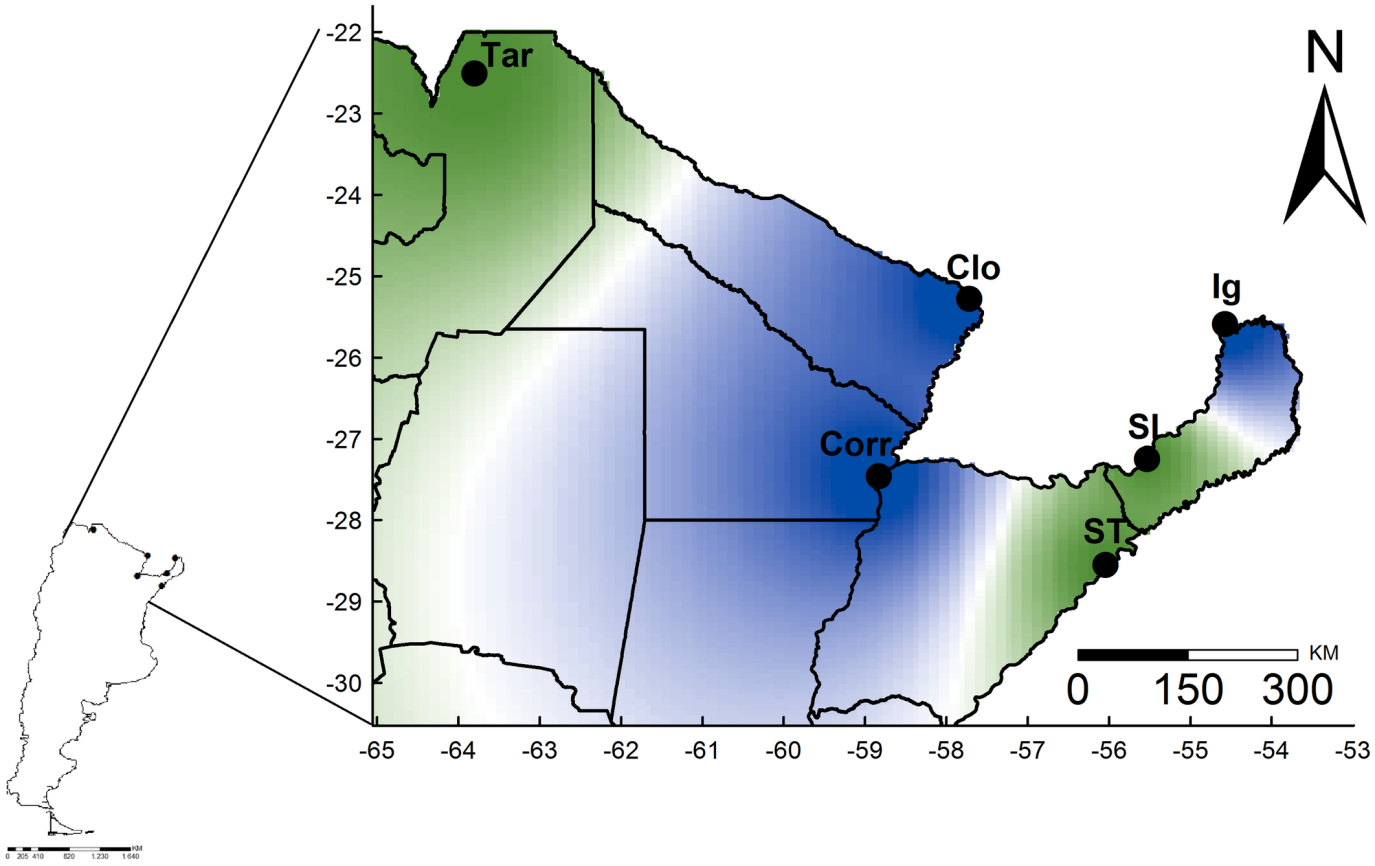
Spatial distribution of Argentinian *Lutzomyia longipalpis* genetic clusters inferred with Geneland. The highest membership values are in green (cluster 1) and blue (cluster 2) to a low PP (light green and light blue to white). Cluster 1: Tartagal (Tar), Santo Tomé (ST), and San Ignacio (SI); Cluster 2: Corrientes city (Corr), Clorinda (Clo) and Puerto Iguazú (Ig). This map was generated using QGIS v.2.6.1 [61].

Geneland analysis (integrating genetic and spatial information) revealed two main genetic clusters (PP = 0.9, K = 2). Each cluster included three sites: Cluster 1 with Tartagal, Santo Tomé, and San Ignacio, and Cluster 2 with Puerto Iguazú, Clorinda, and Corrientes city (Fig 2). According to the AMOVA analysis, differences between the geographic clusters explained 30.16%, whereas inter- and intrapopulation differences explained 12.13% (*p* < 0.0009) and 57.72% (*p* < 0.0001) of the variation, respectively.

### Phylogeographic analysis using the ND4 fragment

**Fig 3:**
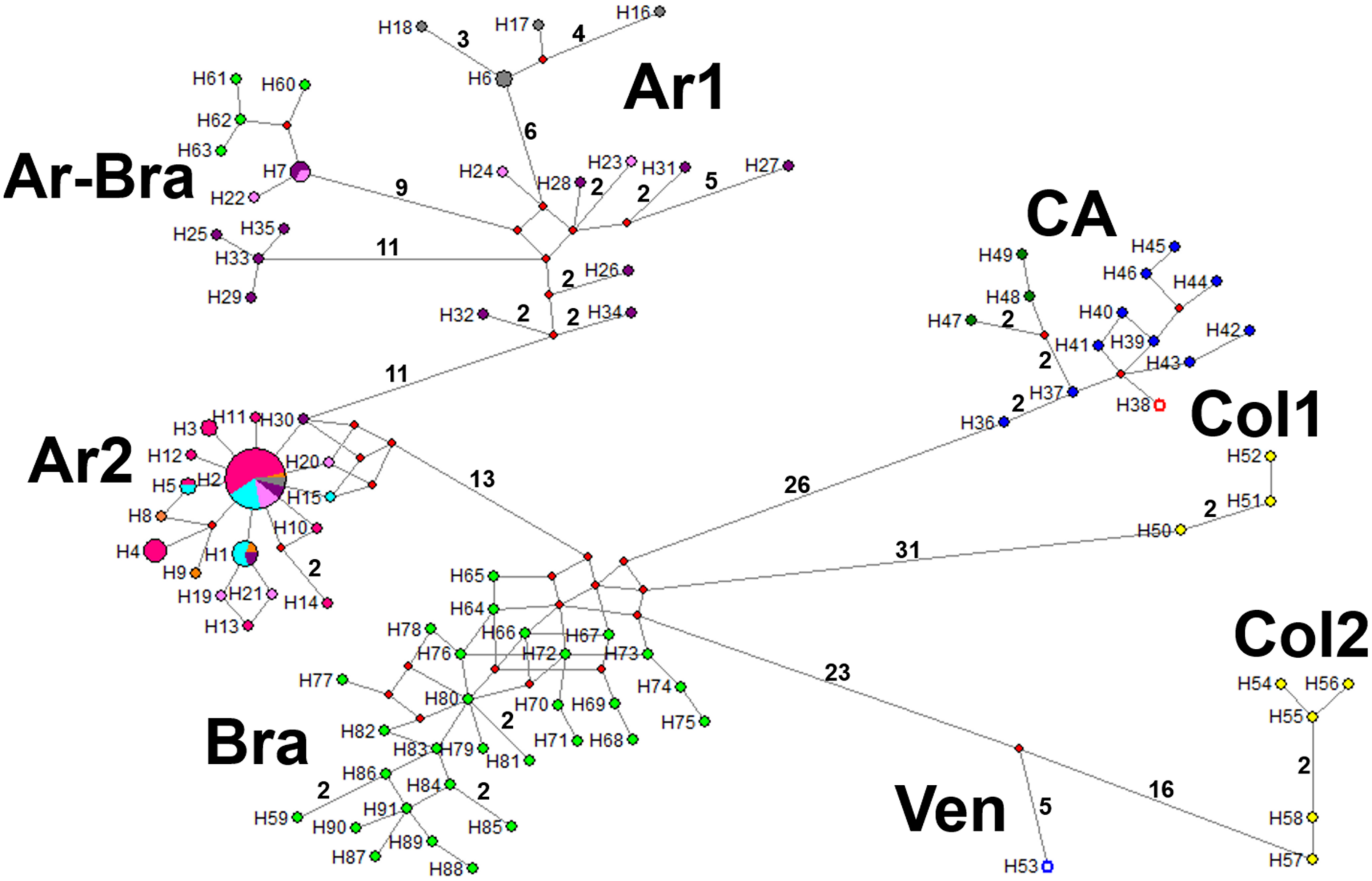
Median-joining haplotype network of the *Lutzomyia longipalpis* complex based on 618 nucleotides of the ND4 gene. The circle size corresponds to the frequency of each haplotype. Missing haplotypes are shown as red circles. Each line connecting haplotypes represents one mutational step, whereas numbers along the lines are total number of mutational steps between haplotypes. Colours indicate: orange = Clorinda; fuchsia = Corrientes city; turquoise = Puerto Iguazú; grey = San Ignacio; pink = Santo Tomé; purple = Tartagal; blue = Honduras; white with red outline = Costa Rica; dark green = Guatemala; yellow = Colombia; white with blue outline = Venezuela; green = Brazil.

**Fig 4:**
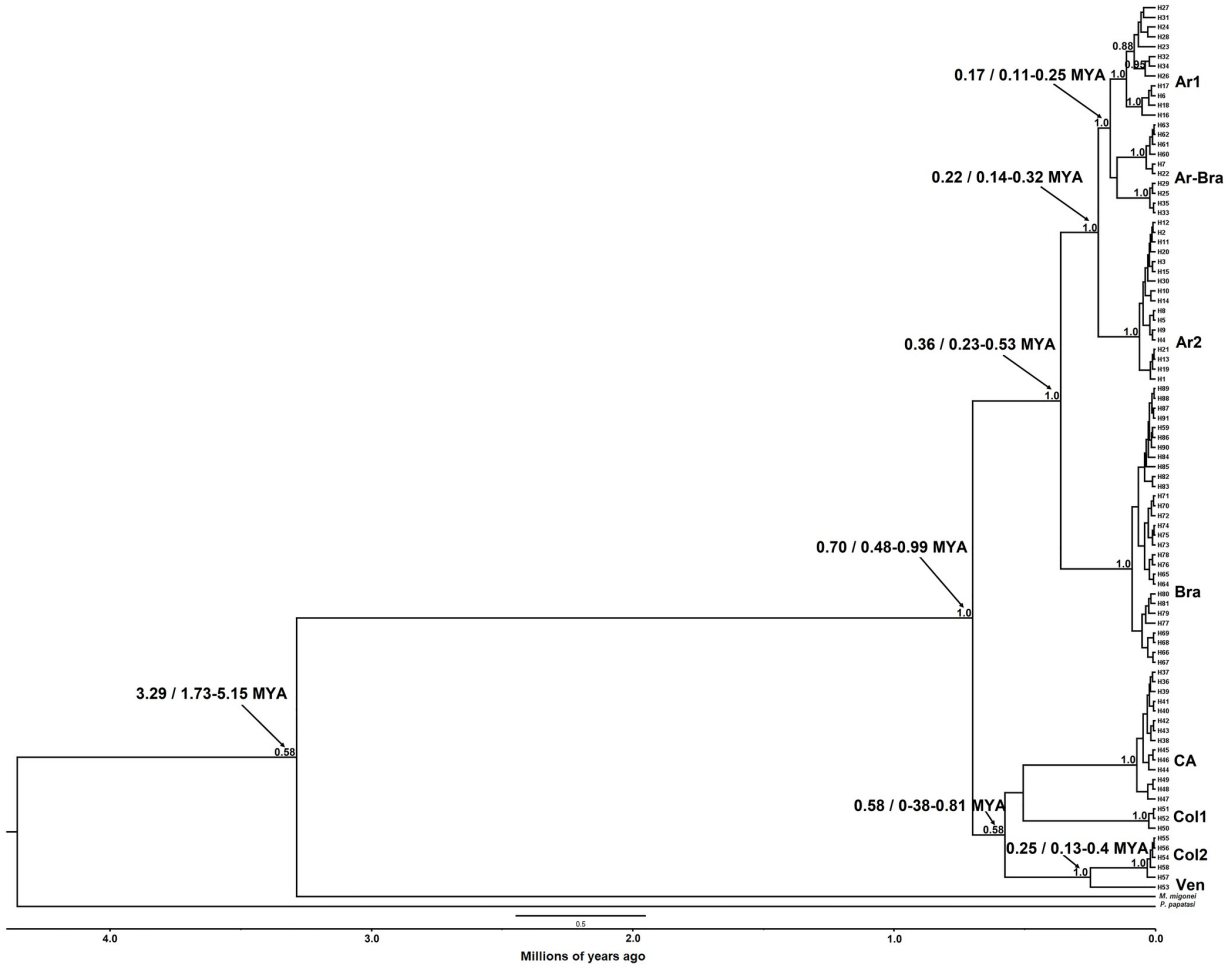
Calibrated maximum-clade-credibility tree for the *Lu*. *longipalpis* complex using the ND4 fragment. The HKY + G model was used for a 618 base pair dataset of the ND4 gene. Numbers above each branch represent PP obtained in the BI (≥ 0.95). *M*. *migonei* and *P*. *papatasi* were used as outgroup. The scale bar represents the line of time in million years ago (MYA) and the numbers indicated by arrows show the time estimate and the 95% HPD.

A total of 91 haplotypes of *Lu*. *longipalpis* reported from seven Latin American countries were included in ND4 phylogeographic analyses (Guatemala, Honduras, Costa Rica, Venezuela, Colombia, Brazil and Argentina) (S1 Table). The Median-joining network identifies at least eight haplogroups, each separated by various mutational steps (BI results were also taken into account for the definition of these haplogroups, Figs 3 and 4). In the Ar1 haplogroup there are 12 haplotypes corresponding to San Ignacio, Santo Tomé and Tartagal specimens, which coincide with Cluster 1 from Geneland analyses. In the Ar-Bra haplogroup, H7 (from both Tartagal and Santo Tomé) and H22 (from Santo Tomé) are grouped with haplotypes from Jacobina and Lapinha in Brazil; there are at least two mutational steps between these haplotypes. The Ar2 haplogroup only includes haplotypes from Argentinian sites, H2 being the most prevalent and shared by all six sites from this study. Haplotypes H15, H20, and H30 are separated by at least 17 mutational steps from the Bra haplogroup. This latter haplogroup includes haplotypes exclusively from Sobral and Pernambuco, Brazil. The haplogroup from Central American (CA) (Honduras, Guatemala, and Costa Rica) is more related to the Bra haplogroup than to the two Colombian haplogroups (Col1 and Col2). The Ven haplogroup (only H53) is more related to haplotypes within the Col2 haplogroup. The two Colombian haplogroups (Col 1 with haplotypes from Giron and Col2 with haplotypes from Neiva and El Callejón) are separated by at least 71 mutational steps (Fig 3, for details of haplotypes see S1 Table).

The BI for ND4 phylogeographic analysis was constructed using the HKY + G model as the most appropriate for the data (-lnL = 2913.8833; BIC = 7035.9464) with gamma of 0.1510. The tree revealed eight haplogroups with maximum support for two major clades (PP = 1.0; Fig 4). One of the major clades is composed of Ar1 (Argentina), Ar-Bra (Argentina-Brazil), Ar2 (Argentina), and Bra (Brazil) haplogroups. The other major clade includes haplogroups from CA (Guatemala, Honduras and Costa Rica), and the three northern South America haplogroups: Col1 (Colombia), Col2 (Colombia), and Ven (Venezuela). Divergence of the *Lu*. *longipalpis* complex from the most recent common ancestor (MRCA) was 0.70 MYA (95% HPD interval = 0.48–0.99 MYA). Divergence of the clade that includes Ar1, Ar-Bra, Ar2 and Bra haplogroups is estimated at 0.36 MYA (95% HPD interval = 0.23–0.53 MYA), and that of the other major clade that includes CA, Col1, Col2, and Ven, 0.58 MYA (95% HPD interval = 0.38–0.81 MYA. The MRCA of Argentinian populations is estimated at 0.22 MYA (95% HPD interval = 0.14–0.32 MYA). Net nucleotide substitutions (*Da*) between haplogroups ranged from 1.2% to 7.5%, while the pairwise F_ST_ between haplogroups was high, with genetic differentiation ranging from 0.468 to 0.966 (Table 3).

**Table 3:** Nucleotide divergence (*Da*) of the ND4 fragment measured as the number of net nucleotide substitutions for *Lu*. *longipalpis* haplogroups (see Results) using the Jukes and Cantor correction (below diagonal), and pairwise F_ST_ comparisons between haplogroups (above diagonal).

	**Ar1 Haplogroup**	**Ar-Bra Haplogroup**	**Ar2 Haplogroup**	**Bra Haplogroup**	**CA Haplogroup**	**Col1 Haplogroup**	**Col2 Haplogroup**
**Ar1 Haplogroup**		0.468***	0.698***	0.801***	0.9***	0.889***	0.888***
**Ar-Bra Haplogroup**	0.012 ± 0.003		0.722***	0.82***	0.895***	0.88***	0.906***
**Ar2 Haplogroup**	0.018 ± 0.002	0.02 ± 0.003		0.849***	0.927***	0.953***	0.955***
**Bra Haplogroup**	0.03 ± 0.003	0.033 ± 0.003	0.03 ± 0.002		0.903***	0.917***	0.933***
**CA Haplogroup**	0.064 ± 0.007	0.062 ± 0.008	0.056 ± 0.005	0.05 ± 0.004		0.929***	0.941***
**Col1 Haplogroup**	0.071 ± 0.016	0.069 ± 0.018	0.07 ± 0.013	0.057 ± 0.008	0.06 ± 0.012		0.966***
**Col2 Haplogroup**	0.066 ± 0.013	0.076 ± 0.016	0.073 ± 0.011	0.067 ± 0.008	0.068 ± 0.011	0.075 ± 0.024	

Statistical significance ***p´ < 0.01802 after Holm-Bonferroni sequential correction.

### Phylogeographic analysis using the cyt *b* fragment

**Fig 5:**
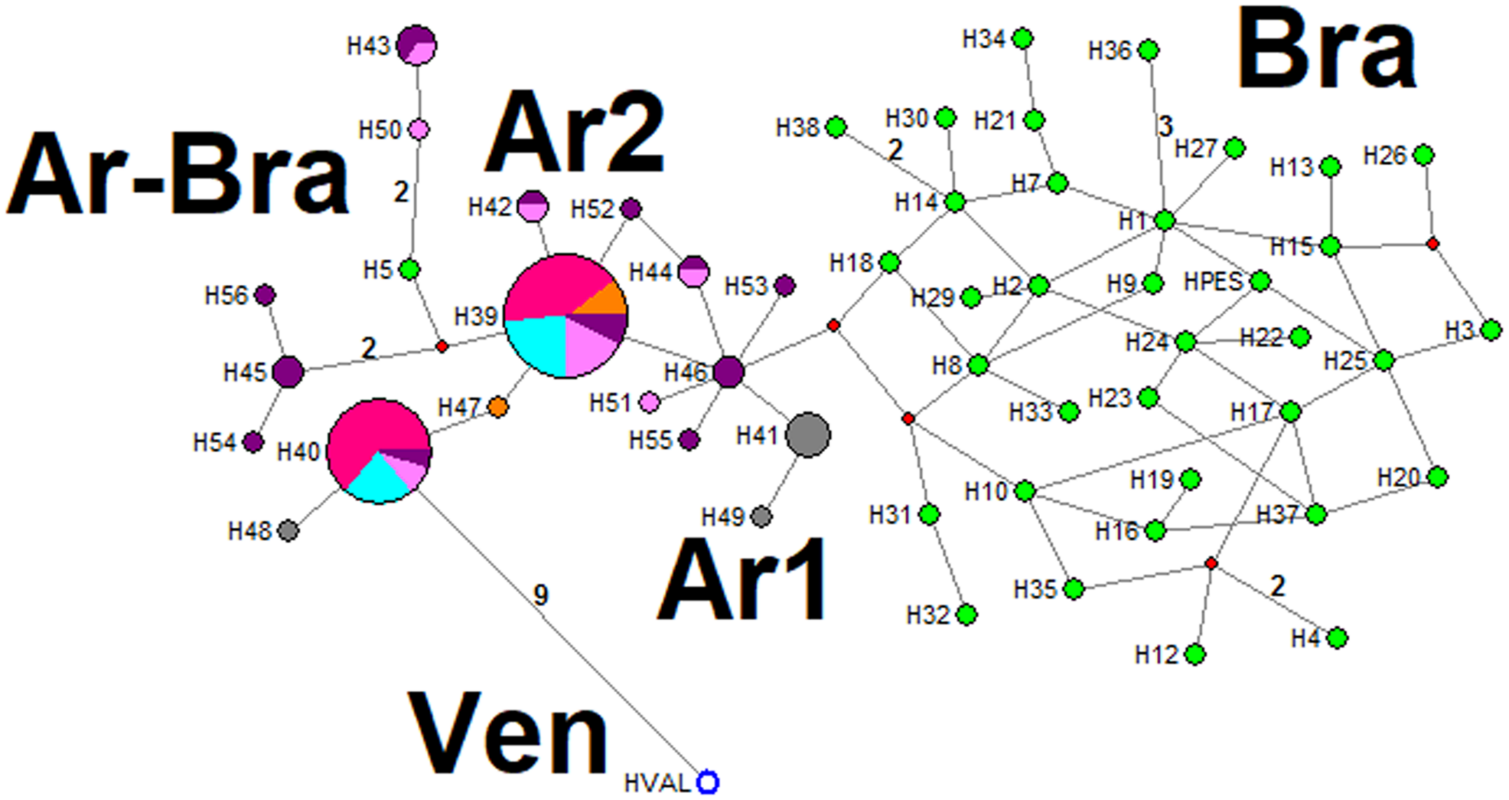
Median-joining haplotype network of the *Lu*. *longipalpis* complex based on 261 nucleotides of the 3´ region of the cyt *b* gene. The circle size corresponds to the frequency of each haplotype. Intermediary haplotypes (missing intermediate haplotypes) are shown as red circles. Each line connecting haplotypes represents one mutational step, whereas numbers along the lines are total number of mutational steps between haplotypes. Colours indicate: orange = Clorinda; fuchsia = Corrientes city; turquoise = Puerto Iguazú; grey = San Ignacio; pink = Santo Tome; purple = Tartagal; white with blue outline = Venezuela; green = Brazil.

**Fig 6:**
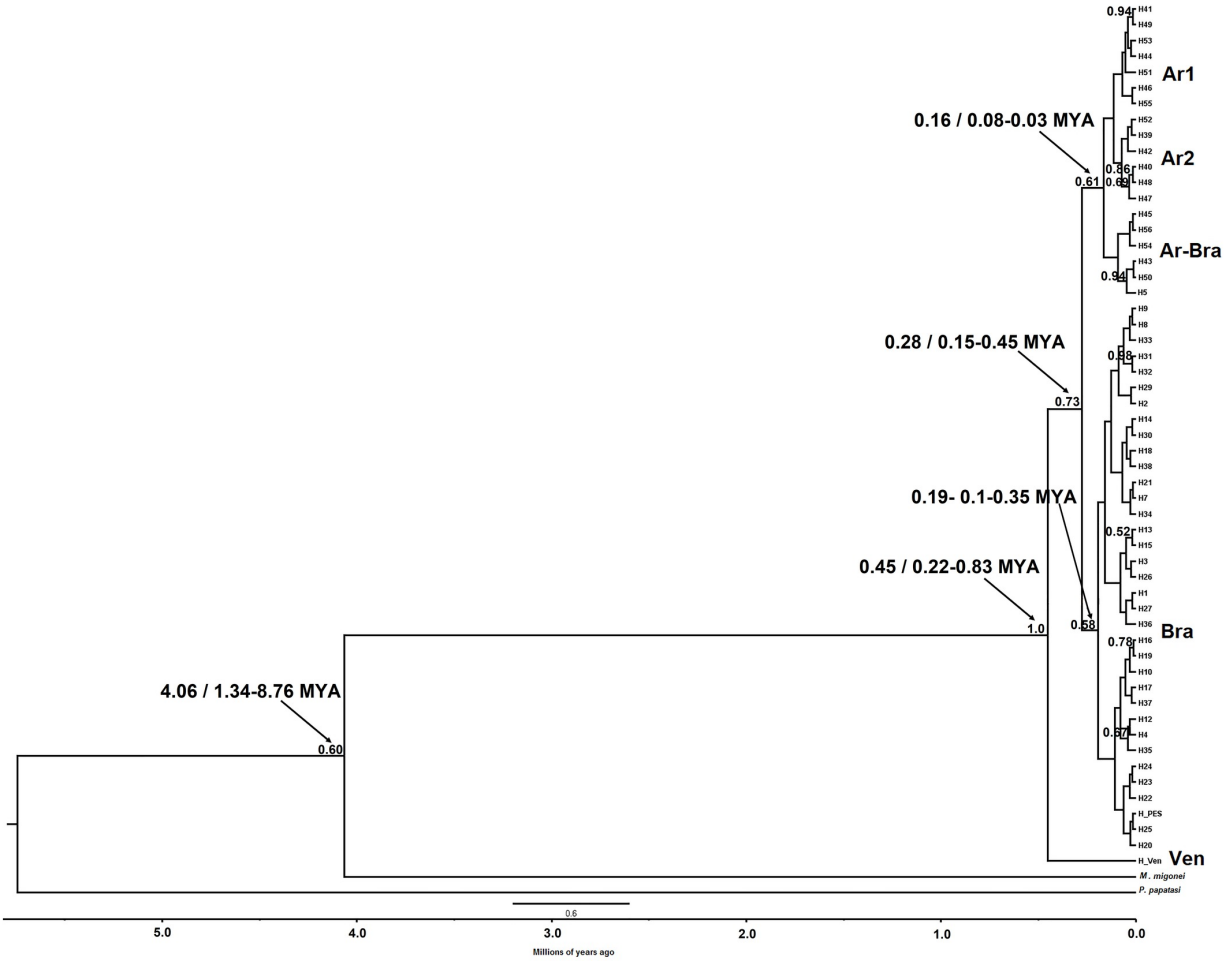
Calibrated maximum-clade-credibility tree for the *Lu*. *longipalpis* complex using the 3´ region of the cyt *b* gene. The HKY + G model was used for a 261 base pair dataset. Numbers above each branch represent PP from the BI (≥ 0.95). *M*. *migonei* and *P*. *papatasi* were used as outgroup. The scale bar represents the line of time in million years ago (MYA) and the numbers indicated by arrows show the time estimate and the 95% HPD.

Phylogeographic analyses using the cyt *b* fragment included 58 haplotypes of *Lu*. *longipalpis* from Venezuela, Brazil, and Argentina (S1 Table). The Median-joining network, had few mutational steps separating haplotypes, identifying five haplogroups (BI was also taken into account for haplogroup definition; Figs 5 and 6). The Bra haplogroup has a difference of two mutational steps with H46/Tartagal/Argentina (Ar1 haplogroup), while the Ar1 haplogroup has haplotypes only from Tartagal, Santo Tomé, and San Ignacio. The Ar2 haplogroup has cyt *b* haplotypes from all six Argentinian sites, the most frequent haplotype being H39 (present in five sites), although this has only a two mutational step difference with H5 (Brazil). The Ar-Bra haplogroup has haplotypes from Tartagal, Santo Tomé and from Juazeiro (Brazil). The single haplotype from Altagracia de Orituco, Venezuela, has a 9 mutational step difference with other frequent haplotypes shared with four Argentinian populations (H40) (Fig 5).

The BI from cyt *b* sequences was constructed using the HKY + G model as the most appropriate for the data (-lnL = 1073.7679; BIC = 2793.0201) with gamma 0.1910. Two major clades were identified, with PP ranging from 0.6 to 1.0: a) one with the Ar1, Ar2, Ar-Bra and Bra haplogroups, and b) the clade with the single haplotype from Venezuela. One haplotype from Juazeiro (H5/Brazil) grouped with Argentinian haplotypes into the Ar-Bra haplogroup (Fig 6). Divergence of the complete *Lu*. *longipalpis* complex from the MRCA was estimated at 0.45 MYA using the cyt *b* fragment (95% HPD interval = 0.22–0.83 MYA). The MRCA for the Argentinian and Brazilian haplogroups was estimated at 0.28 MYA (95% HPD interval = 0.15–0.45 MYA). Net nucleotide substitutions (*Da*) between haplogroups varied from 0.4%–4.7%, while the pairwise F_ST_ between haplogroups was high, genetic differentiation ranging from 0.381 to 0.521 (Table 4).

**Table 4:** Nucleotide divergence (*Da*) from 3´ cyt *b* fragment measured as the number of net nucleotide substitutions between *Lu*. *longipalpis* haplogroups (see Results) using the Jukes and Cantor correction (below diagonal), and pairwise F_ST_ comparisons between *Lu*. *longipalpis* haplogroups (above diagonal).

	**Ar1 Haplogroup**	**Ar2 Haplogroup**	**Ar-Bra Haplogroup**	**Bra Haplogroup**
**Ar1 Haplogroup**		0.39***	0.504***	0.419***
**Ar2 Haplogroup**	0.004 ± 0.003		0.402***	0.381***
**Ar-Bra Haplogroup**	0.01 ± 0.004	0.007 ± 0.004		0.521***
**Bra Haplogroup**	0.01 ± 0.002	0.009 ± 0.002	0.016 ± 0.003	

Statistical significance ***p´ < 0.00238 after Holm-Bonferroni sequential correction.

## Discussion

The two mitochondrial fragments from the ND4 and cyt *b* genes identified high overall haplotype diversity, but relatively low nucleotide diversity and nucleotide polymorphism for *Lu*. *longipalpis* in Argentinian populations. Similar indices for the ND4 gene were reported by Soto *et al*. [16] in *Lu*. *longipalpis* populations from Honduras, Central America (Honduras + Guatemala) and Colombia. Likewise, Coutinho-Abreu *et al*. [20] using the 3´ of the cyt *b* gene from nine Brazilian *Lu*. *longipalpis* populations reported similarly low nucleotide diversity, but higher haplotype diversity. Variability in *Lu*. *longipalpis* is similar to that within other species having wide distributions such as *P*. *papatasi* in the Old World using the ND4 gene [38]. A greater number of ND4 haplotypes were found (*Nh* = 35), than that for the 3´ of cyt *b* gene (*Nh* = 18) in the present study. The populations with highest genetic diversity, using both markers, were Tartagal, Santo Tomé, and San Ignacio. Previous studies have demonstrated that in general, older established populations have higher genetic diversity, which could be related in part to a relatively constant population size [82]. Neutrality indices were not significant for the three populations, which coincides with little change in population size. However, Tartagal had contradictory results, since the cyt *b* neutrality test (*Fs* = -5.598) and its mismatch distribution were consistent with an excess of low-frequency haplotypes, both characteristic of relatively recent population expansion, or of selective sweep/hitch-hiking [83]. In contrast, neutrality tests for the ND4 in Tartagal were not significant and the mismatch distribution was multimodal, which is consistent with demographic equilibrium [84]. Both fragment mismatch distributions from Santo Tomé and San Ignacio indicate that few individuals share each haplotype, which along with high haplotype diversity and a large number of unique haplotypes, may indicate that their populations were historically established.

In comparison, Corrientes city, Puerto Iguazú, and Clorinda had lower genetic diversity, which may be the result of a reduced effective population size and capacity for dispersal (causing loss of genetic diversity and increase of differentiation among the populations). These two features would favor genetic drift, as was proposed for *Lu*. *cruciata* [43], *Lu*. *anduzei* [44], and *Lu*. *olmeca olmeca* [45]. Additionally, populations with low haplotype and nucleotide diversity may have recently experienced prolonged or severe bottlenecks [85]. Results from both gene fragments are contradictory from Corrientes city, the neutrality test with cyt *b* suggesting an important population size reduction (*D* = 2.083, *p* < 0.05, and positive *Fs*), but the ND4 indicating population expansion (significant and negative *Fs* and unimodal mismatch distribution). A greater sampling effort will be required for Puerto Iguazú, Corrientes city, and Clorinda, from which too few haplotypes were identified for analyses and comparisons. These populations should be continuously monitored to analyze the impacts of environmental factors (abiotic, biotic, fragmentation) and healthcare or agricultural interventions (insecticides) over time. Landscape modification or fragmentation caused by anthropogenic actions (e.g. agricultural practices, deforestation) has been shown to be associated with the loss of genetic diversity in *Lu*. *cruciata* [43], *Lu*. *gomezi* [51], *Lu*. *anduzei* [44], and *P*. *papatasi* [39].

Argentinian *Lu*. *longipalpis* populations have high global genetic differentiation, with both markers, perhaps associated with the species´ reduced dispersal capacity. Sandfly dispersal depends on several factors, including average flight distance, wind speed and variation, and distance to resources [86]. It also depends on landscape heterogeneity (abiotic and biotic conditions), which influence female host, resting, mating, and egg laying site selection [87]. Phlebotomines are generally poor fliers, with movement restricted to short, flight-assisted hopping. *Lu*. *longipalpis* flight range is from 1 m to 500 m, but no more than 1,000 m around their breeding sites [86]. This flight range limitation would lead to rapid local population differentiation, due to genetic drift or to population fragmentation [43, 44, 51]. In Argentina, two specific *Lu*. *longipalpis* genetic clusters were identified. Given shared haplotypes in Tartagal and Santo Tomé, cluster 1 may have occurred via a colonization event from Brazil (Ar-Bra haplogroup) or from the Bolivia-Brazil-Paraguay Gran Chaco eco-region, which is associated with sporadic cases of rural human VL [6]. Curiously, there are no sequences of *Lu*. *longipalpis* from these latter countries in GenBank. In the case of Santo Tomé, the most likely route of entry to Argentina is São Borja, Brazil, where an outbreak of VL was reported in 2008 [88]. A similar situation could have occurred with rapid dispersal of *Lu*. *longipalpis*-VL and urban colonization in Campo Grande (Mato Grosso do Sul-Brazil). Subsequent colonization may have occurred via Paraguay and the Argentinian border areas of Clorinda and Posadas. In the present study, we detected genetic similarities among the populations of Clorinda, Corrientes city and Puerto Iguazú (these three populations share several haplotypes and low genetic differentiation), which is in agreement with rapid invasive scenarios of urban *Lu*. *longipalpis*-VL [52, 89].

Bayesian inference using the cyt *b* gene was not well supported, probably due to its low substitution rate [44]. However, too few haplogroups may have been analyzed (Argentinian, Brazilian and Venezuelan), and hence analysis of a larger sample set is recommended. The ND4 gene inference, in contrast, strongly supports monophyly for the *Lu*. *longipalpis* complex. Currently, the exact number of sibling species in the complex remains tentative, due to its broad distribution and absence of sufficient analyses. Soto *et al*. [16] identified four clades using the ND4 gene (Brazil, Central America, and laboratory colony population from Colombia and Venezuela), and similarly, Arrivillaga *et al*. [18, 19] using the COI gene also identified four clades (Laran: Venezuela; cis-Andean: Venezuela, Colombia, northern Brazil; trans-Andean: Venezuela, Colombia, Central America; and Brazilian: Brazil). Analyses of the ND4 sequences from the present study, in addition to those reported by Soto *et al*. [16] and Sonoda [42], identified eight haplogroups: Ar1 (Argentina), Ar2 (Argentina), Ar-Bra (Argentina, Brazil), Bra (Brazil), CA (Guatemala, Honduras, Costa Rica), Col1 (Colombia), Col2 (Colombia) and Ven (Venezuela). The latter haplogroup is interpreted with caution since only one sequence was analyzed despite the MRCA support with 1.0 PP. These eight haplogroups are highly distant by a minimum of several mutational steps, and they have high pairwise F_ST_ and nucleotide divergence, suggesting negligible gene exchange, similar to that suggested in a previous study [16]. This high genetic differentiation among haplogroups may potentially be due to vicariance, and/or climatic tolerance limits, in addition to low dispersal capacity. Geographic and/or climatic barriers have already been associated with diversification not just for *Lu*. *longipalpis* [16, 20, 42], but also for *Lu*. *whitmani* [48, 49], *Lu*. *cruciata* [43], *Lu*. *gomezi* [51], *Lu*. *umbratillis* [90], and *P*. *papatasi* [91].

The present study provides evidence that there are at least three *Lu*. *longipalpis* haplogroups in Argentina. Salomón *et al*. [33] were the first to provide evidence that Argentinian populations may be sibling species to those reported from the northeast and southeast of Brazil [29, 92, 93]. The present study documents significantly high genetic differentiation between the Argentinian and Brazilian haplogroups, despite the Ar-Bra haplogroup, which groups haplotypes from Argentina (Tartagal and Santo Tomé) and Brazil (Jacobina, Lapinha caves and Juazeiro). This genetic difference contrasts with similar complex-level compounds produced by *Lu*. *longipalpis* from Posadas, Argentina [33], Asunción, Paraguay [53], and many populations from Brazil including Lapinha [54].

Divergence of the *Lu*. *longipalpis* complex from its MRCA occurred approximately 0.70 MYA, and resulted in two principal clades, one located east and south of the Amazon basin, giving rise to the principal South American (SA) haplogroups (Brazil and Argentina), and another north from northern South America (NSA), through the Mesoamerican corridor (CA) to Mexico. These two clades are similar to those reported by Arrivillaga *et al*. [18, 19], although in contrast to those reported by Soto *et al*. [16]. Divergence times indicate that this latter cluster from Mesoamerica and NSA, was the first to diverge (0.58 MYA), significantly earlier than the cluster from east and south SA (0.36 MYA). Secondary divergence between the Col2 and the Ven haplogroups (0.25 MYA) and between Ar2 and Ar-Bra/Ar1 haplogroups (0.22 MYA), occurred on similar time scales. Diversification of all haplogroups occurred after the middle Pleistocene, probably during inter-glacial periods, when landscape fragmentation probably provoked diversification hotspots which conserved high diversity [13]. Extreme climate changes that occurred in the Pleistocene forced adaptation/selection of most biota, including phlebotomine sandflies, to resulting biotic and abiotic conditions [94]. Sandflies may have been subjected to local cycles of dry and cold periods, taking refuge and adapting to the more permanent humid resting fragments, which allowed these haplogroups and diversity to evolve [13, 95]. Indeed, plant species’ distribution shifts resulting from climate variation during the Pleistocene, have also been associated with changes in *Lu*. *longipalpis* complex diversification [18, 95, 96]. Arrivillaga *et al*., [18, 19] suggest that divergence was probably a result of vicariance events that occurred throughout the late Pliocene and Pleistocene (e.g. Andean orogeny). However, recent analyses indicate a highly conserved geographic coverage of the ecological niche of *Lu*. *longipalpis* from the Last Glacial Maximum (LGM) in northern Mesoamerica (88.1%), the species complex´ northern limit [13]. Hence, genetic diversity is highest in the most temporally conserved landscapes. This hypothesis can now be analyzed between and within the major *Lu longipalpis* haplogroup clades.

This is the first report on the genetic diversity of *Lu*. *longipalpis* from Argentinian populations, which has high genetic differentiation, two genetic clusters, and three haplogroups. Phylogeographic results provide evidence for a high level of divergence among the eight haplogroups identified for the *Lu*. *longipalpis* complex using ND4. Finally, the findings represent only the first stage of future studies required to include a more balanced sampling across *Lu*. *longipalpis* distributions and a greater number of samples not only within Argentina, but in all continental subregions. Multilocus genetic analyses will also be required in order to more completely understand evolutionary processes in this important vector species complex, and the impact of environmental change on vector transmission risk of VL. The understanding of the biological and evolutionary aspects of this species complex at the micro and macro-evolutionary levels are central to understanding the interplay between vector capacity, vectorial competence for different *Leishmania* parasites, urban colonization potential (micro-environment adaptation), demographic aspects of *Leishmania* transmission, and the clinical expression of disease [30, 55].

## Supporting information

S1 Table Origin of *Lutzomyia longipalpis* samples included in analyses. (XLSX)

S1 Fig Mismatch distributions based on 618 bp of the ND4 gene from six populations of *Lu*. *longipalpis* from Argentina. The black lines are observed distribution, the dotted line indicates the distribution simulated under a sudden expansion model. The sum of squared deviations (*SSD*) and Harpending’s raggedness index (*r*) and corresponding *p*-values are shown.
(TIF)

S2 Fig Mismatch distributions based on 261 nucleotides of the 3´ region of the cyt *b* gene from six populations of *Lu*. *longipalpis* from Argentina. The black lines are observed distribution, the dotted line indicates the distribution simulated under a sudden expansion model. The sum of squared deviations (*SSD*) and Harpending’s raggedness index (*r*) and corresponding *p*-value are shown.
(TIF)
