# Supplementary figures and images for: Genetic diversity, phylogeography and molecular clock of the Lutzomyia longipalpis complex (Diptera: Psychodidae)
Source: PLoS Negl Trop Dis. 2018 Jul 5;12(7):e0006614. doi: 10.1371/journal.pntd.0006614 (PMC6049954; doi:10.1371/journal.pntd.0006614)

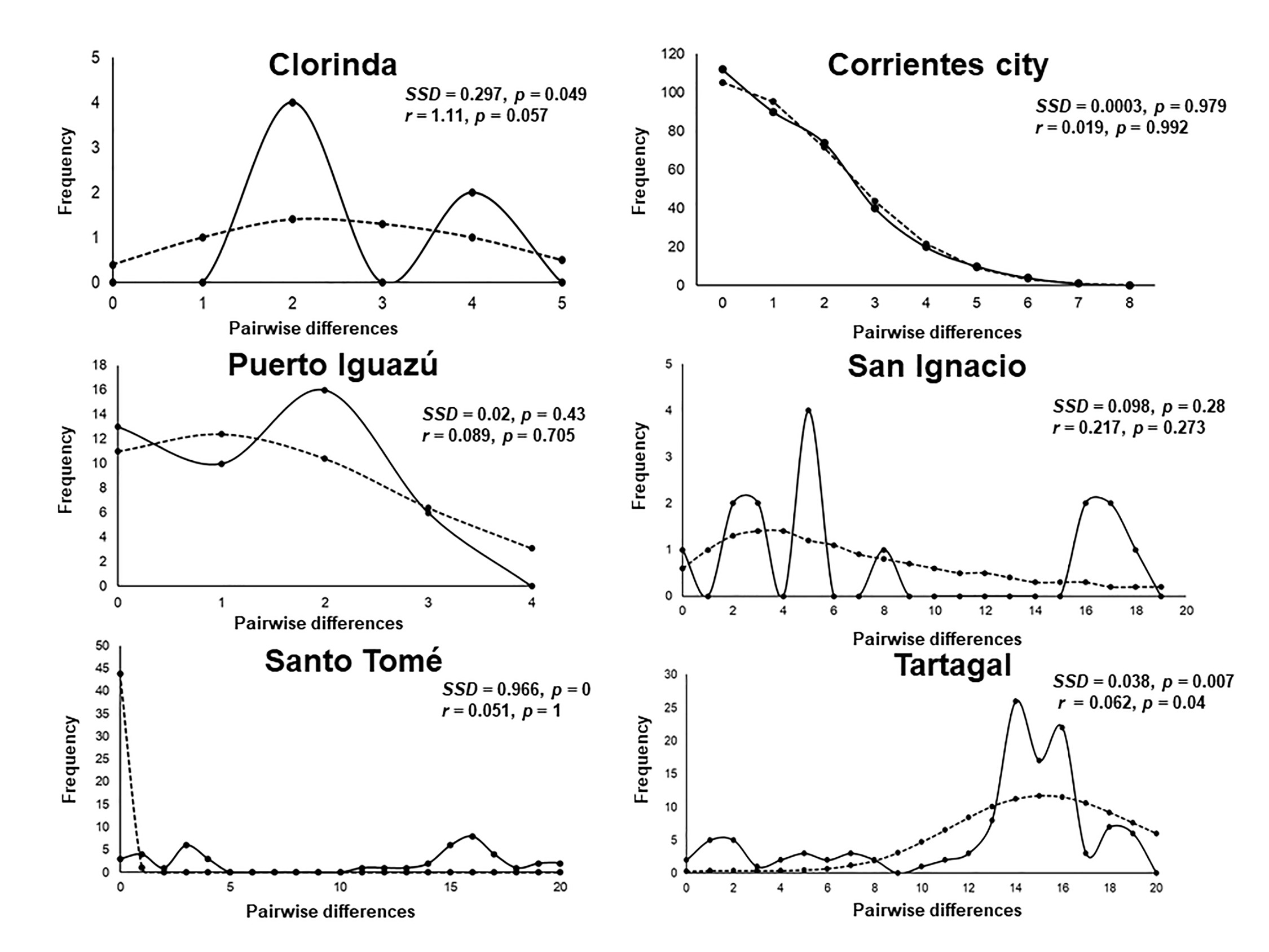

Supplement: S1 Fig — The black lines are observed distribution, the dotted line indicates the distribution simulated under a sudden expansion model. The sum of squared deviations (SSD) and Harpending’s raggedness index (r) and corresponding p-values are shown. (TIF) [file pntd.0006614.s002.tif]

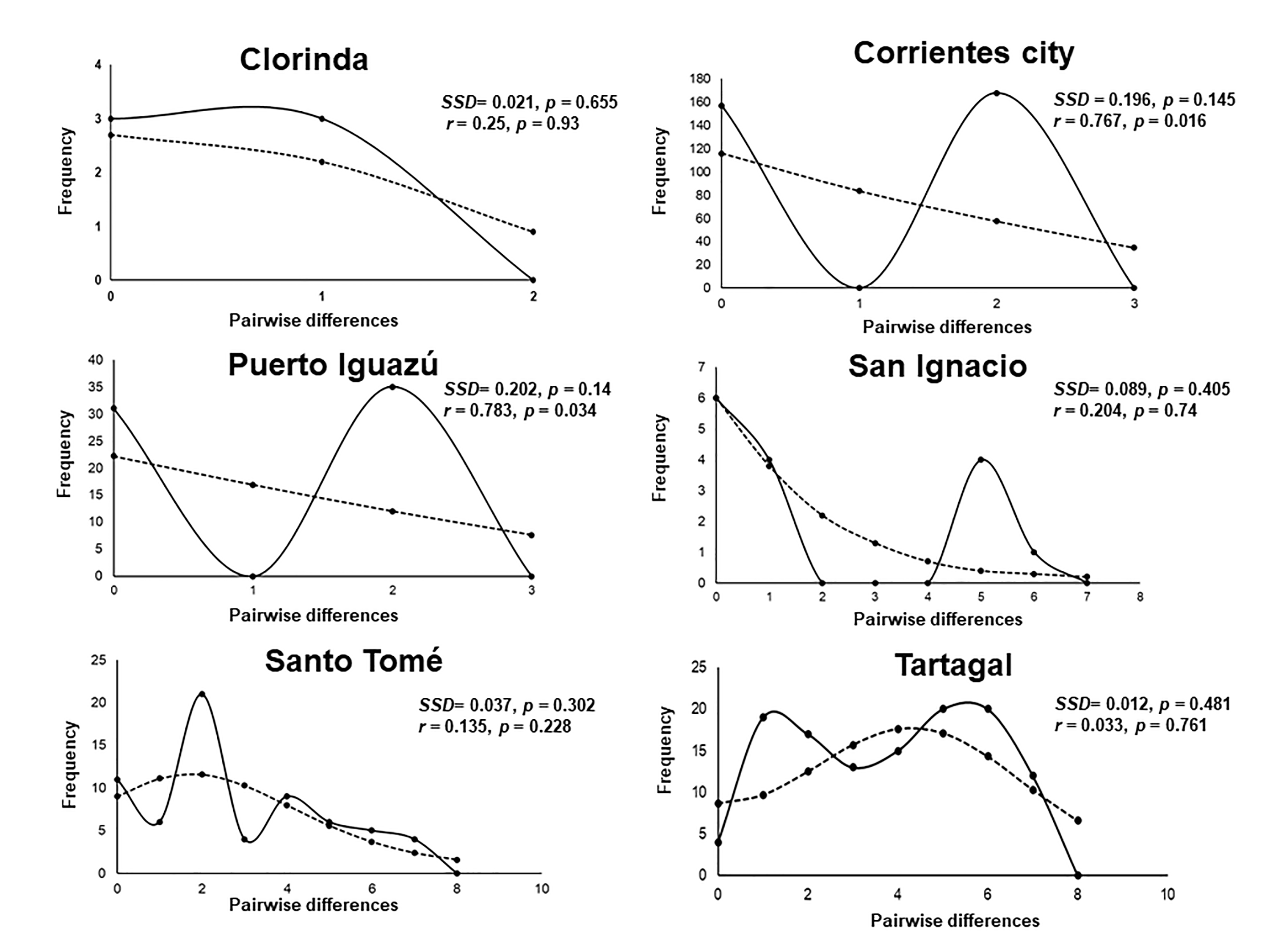

Supplement: S2 Fig — The black lines are observed distribution, the dotted line indicates the distribution simulated under a sudden expansion model. The sum of squared deviations (SSD) and Harpending’s raggedness index (r) and corresponding p-value are shown. (TIF) [file pntd.0006614.s003.tif]
